# Patient involvement in clinical research: a qualitative evaluation of impact

**DOI:** 10.1186/1745-6215-14-S1-O36

**Published:** 2013-11-29

**Authors:** Rachael Gooberman-Hill, Amanda Burston, Erik Lenguerrand, Emma Clark, Emma Johnson

**Affiliations:** 1 University of Bristol, Bristol, UK

## Background

Patients are increasingly involved in design and implementation of clinical studies. Despite checklists for reporting patient involvement, and increasing focus on the impact of patient involvement, evaluations remain scarce. We evaluated impact of patient involvement in an NIHR supported research programme focusing on joint replacement: ‘RESTORE'.

## Context

The RESTORE programme comprises a randomised controlled trial, three pilot trials, a cohort study, qualitative studies and systematic reviews. Since 2010, the patient panel (PEP-R), has inputted into RESTORE. Seventeen patients have been involved in total, 8 were recruited at the time of the evaluation. Through facilitated group sessions, PEP-R provides input into refinement of patient recruitment materials, intervention development, readability of outcome assessment tools, and dissemination of findings. Patients also sit on steering groups and receive training.

## Methods

Impact of patient involvement was evaluated through questionnaires completed by patients and research staff. All participants were asked to identify the impact of PEP-R on themselves and the research. Eight patients and 14 researchers completed the questionnaire. Responses were analysed using qualitative ‘framework' approach.

## Results

Patients described their interest in learning about research. They valued feedback about how PEP-R's input had shaped studies. Researchers identified the benefits of obtaining patients' views on the importance, relevance and feasibility of their projects. They welcomed the opportunity to speak to an interested and knowledgeable group and stressed the importance of early involvement.

## Conclusion

Patients and researchers see patient involvement as beneficial. The model used in PEP-R facilitates patient involvement in the design and implementation of clinical research.

